# Supplementary material for: Acetylation of histones and non-histone proteins is not a mere consequence of ongoing transcription
Source: Nat Commun. 2024 Jun 11;15:4962. doi: 10.1038/s41467-024-49370-2 (PMC11166988; doi:10.1038/s41467-024-49370-2)
Supplement: Supplementary file 1 — Supplementary Information [file 41467_2024_49370_MOESM1_ESM.pdf]

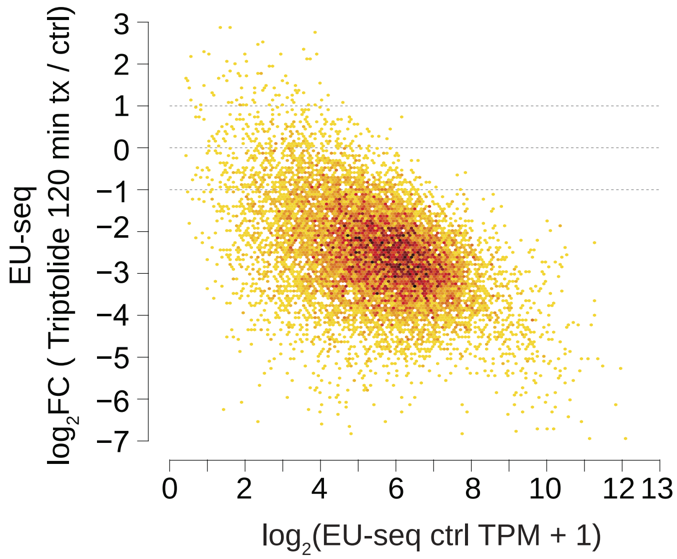

Supplementary Fig. 1. Triptolide acutely inhibits transcription. Shown is the fold change and abundance of transcripts quantified by EU-seq from mESC treated with triptolide 5M for 120min with 2 biological replicates. In both treatments, median relative abundance of transcripts was normalized to mitochondrial transcripts reads.

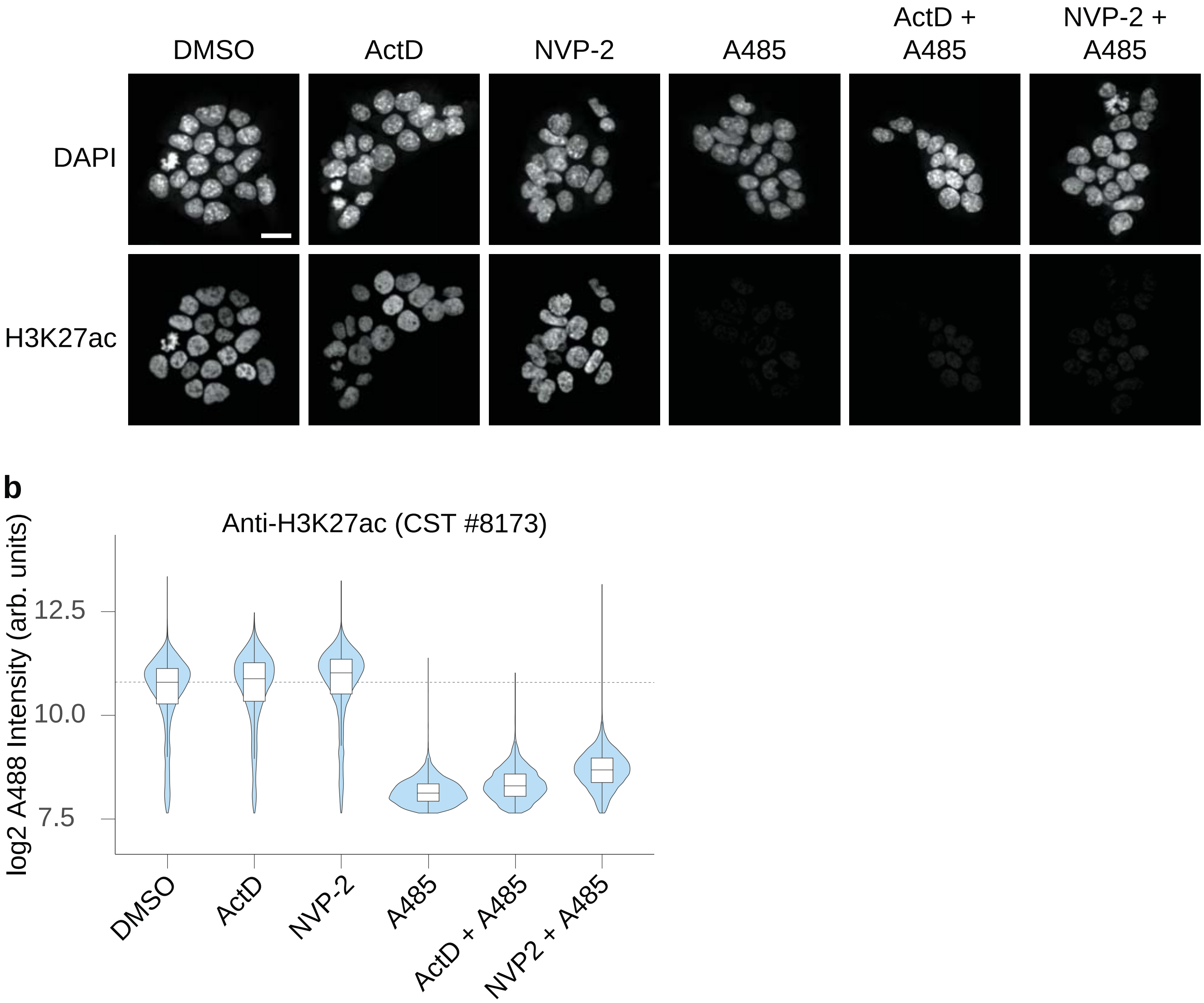

Supplementary Fig. 2. H3K27ac is reduced by CBP/p300 inhibition but not by transcription inhib ition by Actinomycin D or NVP-2 treatment. a Representative images of mESC treated the indicat ed transcription inhibitors or A-485 (concentrations: A-485 10μM, ActD 1μg/mL, NVP-2 1μM). Cel ls were treated with the indicated inhibitors for 2 hours, stained with H3K27ac antibody (Cell Sign aling Technology, clone #D5E4), and analyzed by immunofluorescence. White scale bar is 50μm . b Median nuclear staining intensity relative to the DMSO control for the different combinations of treatments and staining as determined from image-based cytometry analysis of at least 2800 automatically selected cells per combination. The dotted line indicates the median intensity of the DMSO treated cells. Violin plots show the distribution of the data, and the box represent the 25th and 75th percentiles as lower and upper hinges, with the bar within box indicates median. The whiskers show 1.5× IQR.

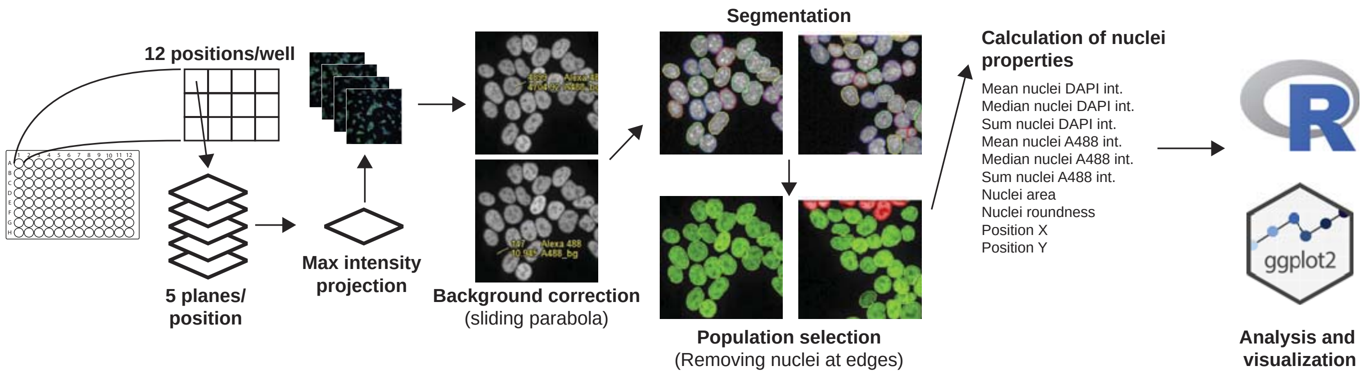

Supplementary Fig. 3. Schematic display of the quantitative image-based cytometry acquisition, and analysis. Each well was imaged at 12 positions and 5 planes per position. Maximum intensity projections of each position were calculated and advanced flatfield, brightfield as well as background corrections applied. Nuclei were identified using the DAPI and nuclei at the border of images were removed. The DAPI and A488 images of the selected population was used to calculate nuclei properties.

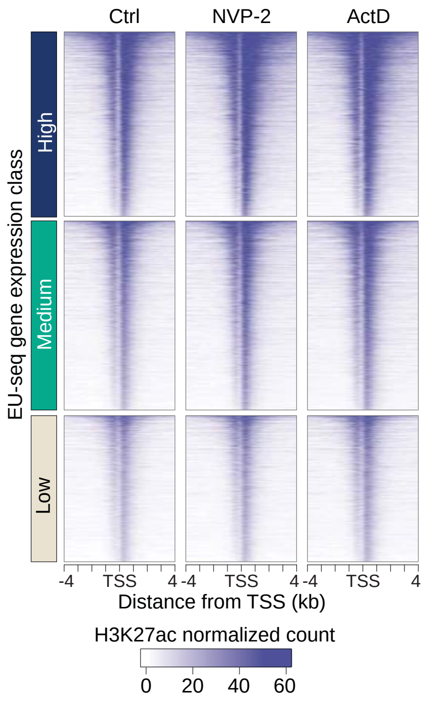

Supplementary Fig. 4. H3K27ac level remain unchanged in promoters of high, medium, and low expressed genes after transcription inhibition. Heatmap of spike-in normalized H3K27ac ChIP signal intensities around TSS regions (+/- 4kb). H3K27ac peak signals overlapping with TSS are classified into High, Middle and Low groups based on the EU-seq TPM values (High: upper tertile, Middle: middle tertile, Low: lower tertile of EU-seq TPM values among expressed genes under control condition in mouse ESC). Within each category, the peaks are sorted by the intensity of H3K27ac signal in control condition. All the ChIP-seq experiments were performed with 2 biological replicates.
