# Supplementary material for: Acetylation of histones and non-histone proteins is not a mere consequence of ongoing transcription
Source: Nat Commun. 2024 Jun 11;15:4962. doi: 10.1038/s41467-024-49370-2 (PMC11166988; doi:10.1038/s41467-024-49370-2)
Supplement: Supplementary file 2 — Reporting Summary [file 41467_2024_49370_MOESM2_ESM.pdf]

nature portfolio

Corresponding author(s): Chunaram Choudhary

Last updated by author(s): May 22, 2024

Reporting Summary

Nature Portfolio wishes to improve the reproducibility of the work that we publish. This form provides structure for consistency and transparency in reporting. For further information on Nature Portfolio policies, see our Editorial Policies and the Editorial Policy Checklist.

Statistics

For all statistical analyses, confirm that the following items are present in the figure legend, table legend, main text, or Methods section.

n/a	Confirmed	
☐	☒	The exact sample size (*n*) for each experimental group/condition, given as a discrete number and unit of measurement
☐	☒	A statement on whether measurements were taken from distinct samples or whether the same sample was measured repeatedly
☐	☒	The statistical test(s) used AND whether they are one- or two-sided<br>*Only common tests should be described solely by name; describe more complex techniques in the Methods section.*
☒	☐	A description of all covariates tested
☐	☒	A description of any assumptions or corrections, such as tests of normality and adjustment for multiple comparisons
☐	☒	A full description of the statistical parameters including central tendency (e.g. means) or other basic estimates (e.g. regression coefficient) AND variation (e.g. standard deviation) or associated estimates of uncertainty (e.g. confidence intervals)
☐	☒	For null hypothesis testing, the test statistic (e.g. *F*, *t*, *r*) with confidence intervals, effect sizes, degrees of freedom and *P* value noted<br>*Give P values as exact values whenever suitable.*
☒	☐	For Bayesian analysis, information on the choice of priors and Markov chain Monte Carlo settings
☒	☐	For hierarchical and complex designs, identification of the appropriate level for tests and full reporting of outcomes
☐	☒	Estimates of effect sizes (e.g. Cohen's *d*, Pearson's *r*), indicating how they were calculated

Our web collection on statistics for biologists contains articles on many of the points above.

Software and code

Policy information about availability of computer code

Data collection	No special software was used to collect data.
Data analysis	All analysis were performed in R. No custom code was used for analyzing the data.

For manuscripts utilizing custom algorithms or software that are central to the research but not yet described in published literature, software must be made available to editors and reviewers. We strongly encourage code deposition in a community repository (e.g. GitHub). See the Nature Portfolio guidelines for submitting code & software for further information.

Data

Policy information about availability of data

All manuscripts must include a data availability statement. This statement should provide the following information, where applicable:

- Accession codes, unique identifiers, or web links for publicly available datasets
- A description of any restrictions on data availability
- For clinical datasets or third party data, please ensure that the statement adheres to our policy

The mass spectrometry data have been deposited to the ProteomeXchange Consortium via the PRIDE partner repository with the dataset identifier PXD044009 [https://www.ebi.ac.uk/pride/archive/projects/PXD044009].

Research involving human participants, their data, or biological material

Policy information about studies with human participants or human data. See also policy information about sex, gender (identity/presentation), and sexual orientation and race, ethnicity and racism.

Reporting on sex and gender	*Use the terms sex (biological attribute) and gender (shaped by social and cultural circumstances) carefully in order to avoid confusing both terms. Indicate if findings apply to only one sex or gender; describe whether sex and gender were considered in study design; whether sex and/or gender was determined based on self-reporting or assigned and methods used. Provide in the source data disaggregated sex and gender data, where this information has been collected, and if consent has been obtained for sharing of individual-level data; provide overall numbers in this Reporting Summary. Please state if this information has not been collected. Report sex- and gender-based analyses where performed, justify reasons for lack of sex- and gender-based analysis.*
Reporting on race, ethnicity, or other socially relevant groupings	*Please specify the socially constructed or socially relevant categorization variable(s) used in your manuscript and explain why they were used. Please note that such variables should not be used as proxies for other socially constructed/relevant variables (for example, race or ethnicity should not be used as a proxy for socioeconomic status). Provide clear definitions of the relevant terms used, how they were provided (by the participants/respondents, the researchers, or third parties), and the method(s) used to classify people into the different categories (e.g. self-report, census or administrative data, social media data, etc.) Please provide details about how you controlled for confounding variables in your analyses.*
Population characteristics	*Describe the covariate-relevant population characteristics of the human research participants (e.g. age, genotypic information, past and current diagnosis and treatment categories). If you filled out the behavioural & social sciences study design questions and have nothing to add here, write "See above."*
Recruitment	*Describe how participants were recruited. Outline any potential self-selection bias or other biases that may be present and how these are likely to impact results.*
Ethics oversight	*Identify the organization(s) that approved the study protocol.*

Note that full information on the approval of the study protocol must also be provided in the manuscript.

Field-specific reporting

Please select the one below that is the best fit for your research. If you are not sure, read the appropriate sections before making your selection.

☒ Life sciences ☐ Behavioural & social sciences ☐ Ecological, evolutionary & environmental sciences

For a reference copy of the document with all sections, see nature.com/documents/nr-reporting-summary-flat.pdf

Life sciences study design

All studies must disclose on these points even when the disclosure is negative.

Sample size	We did not predetermine a sample size beforehand. The treatment involved prolonged exposure to transcriptional inhibitors, which was anticipated to yield dramatic changes in the observed feature. Given the robust nature of the expected outcomes, even a small sample size was presumed sufficient to detect significant effects.
Data exclusions	The first replicate of the acetylome was excluded due to the low depth of measurements. All attempts to repeat the experiment were successful.
Replication	We generally performed at least two independent biological replicates per condition. For ActinomycinD and NVP2 proteomic experiments six replicates were performed.
Randomization	No randomization was performed since it was not relevant to the study design.
Blinding	No blinding was performed since it was not relevant to the study since no clinical samples were involved. All experiments were conmducted in cell lines and blinding was not necessary as the data were generated by digital read-outs.

Reporting for specific materials, systems and methods

We require information from authors about some types of materials, experimental systems and methods used in many studies. Here, indicate whether each material, system or method listed is relevant to your study. If you are not sure if a list item applies to your research, read the appropriate section before selecting a response.

Materials & experimental systems

n/a	Involved in the study
[ ]	[x] Antibodies
[ ]	[x] Eukaryotic cell lines
[x]	[ ] Palaeontology and archaeology
[x]	[ ] Animals and other organisms
[x]	[ ] Clinical data
[x]	[ ] Dual use research of concern
[x]	[ ] Plants

Methods

n/a	Involved in the study
[ ]	[x] ChIP-seq
[x]	[ ] Flow cytometry
[x]	[ ] MRI-based neuroimaging

Antibodies

Antibodies used

Abcam Recombinant Anti-Histone H3 (acetyl K27) antibody [EP16602] - ChIP Grade (ab177178); Cell Signaling Technology Ubiquityl-Histone H2B (Lys120) (D11) XP® Rabbit mAb #5546; Cell Signaling Technology Acetyl-Histone H3 (Lys27) (D5E4) XP® Rabbit mAb #8173

Validation

Abcam Recombinant Anti-Histone H3 (acetyl K27) antibody [EP16602] - ChIP Grade (ab177178), Cell Signaling Technology Ubiquityl-Histone H2B (Lys120) (D11) XP® Rabbit mAb #5546 and Cell Signaling Technology Acetyl-Histone H3 (Lys27) (D5E4) XP® Rabbit mAb #8173 were validated by the manufacturer. No further validation was performed.

Eukaryotic cell lines

Policy information about cell lines and Sex and Gender in Research

Cell line source(s)

ES-E14TG2a cells from Sigma Aldrich.

Authentication

The cell line obtained from a commercial source, and was not authenticated further.

Mycoplasma contamination

Cell lines were not tested for mycoplasma contatmination.

Commonly misidentified lines
(See ICLAC register)

No commonly misidentified cell line was used in this study.

Plants

Seed stocks

Report on the source of all seed stocks or other plant material used. If applicable, state the seed stock centre and catalogue number. If plant specimens were collected from the field, describe the collection location, date and sampling procedures.

Novel plant genotypes

Describe the methods by which all novel plant genotypes were produced. This includes those generated by transgenic approaches, gene editing, chemical/radiation-based mutagenesis and hybridization. For transgenic lines, describe the transformation method, the number of independent lines analyzed and the generation upon which experiments were performed. For gene-edited lines, describe the editor used, the endogenous sequence targeted for editing, the targeting guide RNA sequence (if applicable) and how the editor was applied.

Authentication

Describe any authentication procedures for each seed stock used or novel genotype generated. Describe any experiments used to assess the effect of a mutation and, where applicable, how potential secondary effects (e.g. second site T-DNA insertions, mosiacism, off-target gene editing) were examined.

ChIP-seq

Data deposition

[x] Confirm that both raw and final processed data have been deposited in a public database such as GEO.

[x] Confirm that you have deposited or provided access to graph files (e.g. BED files) for the called peaks.

Data access links
May remain private before publication.

The raw data and genome browser tracks of EU-seq in Triptolide-treated ESC, and H3K27ac ChIP-seq data with or without NVP2- or actinomycin D-treated ESC are available in the NCBI Gene Expression Omnibus (GEO) database under accession code GSE260969 [https://www.ncbi.nlm.nih.gov/geo/query/acc.cgi?acc=GSE260969].

Files in database submission

EU-seq
EUSeq.ESC_TRP_500nM_TC120_EU.CC1_1.fastq.gz
EUSeq.ESC_TRP_500nM_TC120_EU.CC1_2.fastq.gz
ChIP-seq
ESC_Ctrl_TC0_H3K27ac.ab4729_spike.HEK293_CC39_1.fastq.gz

ESC_Ctrl_TC0_H3K27ac.ab4729_spike.HEK293_CC44_1.fastq.gz
ESC_ActD_800nM_TC120_H3K27ac.ab4729_spike.HEK293_CC39_1.fastq.gz
ESC_ActD_800nM_TC120_H3K27ac.ab4729_spike.HEK293_CC44_1.fastq.gz
ESC_NVP2_100nM_TC120_H3K27ac.ab4729_spike.HEK293_CC39_1.fastq.gz
ESC_NVP2_100nM_TC120_H3K27ac.ab4729_spike.HEK293_CC44_1.fastq.gz

Genome browser session
(e.g. UCSC)

Provide a link to an anonymized genome browser session for "Initial submission" and "Revised version" documents only, to enable peer review. Write "no longer applicable" for "Final submission" documents.

Methodology

Replicates

Two biological replicates were performed for all the EU-seq and ChIP-seq experiments.

Sequencing depth

EU-seq
EUSeq.ESC_TRP_500nM_TC120_EU.CC1_1.fastq.gz, total: 35134776, mapped (excluding rRNA, tRNA):15597663, 75bp, single-end
EUSeq.ESC_TRP_500nM_TC120_EU.CC1_2.fastq.gz, total: 31330923, unique: 10659783, 75bp, single-end
ChIP-seq
ESC_Ctrl_TC0_H3K27ac.ab4729_spike.HEK293_CC39_1.fastq.gz, total: 38263280, unique: 28606129, 132bp, single-end
ESC_Ctrl_TC0_H3K27ac.ab4729_spike.HEK293_CC44_1.fastq.gz, total: 97239944, unique: 73909764, 132bp, single-end
ESC_ActD_800nM_TC120_H3K27ac.ab4729_spike.HEK293_CC39_1.fastq.gz, total: 33396885, unique: 25194508, 132bp, single-end
ESC_ActD_800nM_TC120_H3K27ac.ab4729_spike.HEK293_CC44_1.fastq.gz, total: 71560045, unique: 54888075, 132bp, single-end
ESC_NVP2_100nM_TC120_H3K27ac.ab4729_spike.HEK293_CC39_1.fastq.gz, total: 34580851, unique: 26122700, 132bp, single-end
ESC_NVP2_100nM_TC120_H3K27ac.ab4729_spike.HEK293_CC44_1.fastq.gz, total: 59992839, unique: 46599425, 132bp, single-end

Antibodies

The following antibody was used for ChIP-seq.
H3K27ac: manufacturer, Abcam; cat# ab4729, clone# Rabbit polyclonal

Peak calling parameters

Read sequences were aligned to the combined mouse mm10 and human T2T-CHM13v2 genome using bwa meme (version 1.0.4, PMID: 35253835) with soft clipping option for supplementary alignments. Duplicated reads were annotated and removed using Picard toolkit tools (version 2.9.1, "Picard Toolkit." 2019. Broad Institute, GitHub Repository. https://broadinstitute.github.io/picard/; Broad Institute). Low-mapping quality reads (MAPQ < 10) were excluded using samtools (version 1.4)89. Peak calling was conducted using LanceOtron with the default model (wide-and-deep_jan-2021)92. The peaks proximal within 2kb are merged using Bedtools93. Peak heights were calculated using bamCompare94 with the following parameters (centerReads, minMappingQuality 10, bin size 20b, smoothing length 400b, extension of reads to 200b, rpm normalization, and input rpm value is subtracted). The peak summit was defined as the center of the 20b bin at maximum height in each peak region. Poorly enriched peaks of maximum peak height at 20bp bin < 8 reads mapped per million (rpm) or read enrichment at +/- 500bp around peak summit center < 1 rpm after input subtraction were excluded. H3K27ac peaks that were not proximal to ATAC-seq peak summit within 400bp, or whose peak summits were more than 1kb from ATAC-seq peak summit were filtered out.

Data quality

FASTQC was used for quality check of sequencing reads.

Software

Cutadapt, fastqc (0.11.5), STAR(2.6.1a), Bedtools(2.23), HTseq(0.11.1), BWA meme(1.0.4), Picard-tools (2.9.1), samtools(1.4), Lanceotron(20210215), R(4.1.1), DESeq(1.32.0), ChIPseeker(1.28.3), GenomicRanges(1.44.0), deepTools2 (3.5.2)
