# Supplementary material for: Acetylation of histones and non-histone proteins is not a mere consequence of ongoing transcription
Source: Nat Commun. 2024 Jun 11;15:4962. doi: 10.1038/s41467-024-49370-2 (PMC11166988; doi:10.1038/s41467-024-49370-2)
Supplement: Supplementary file 3 — Description of Additional Supplementary Files [file 41467_2024_49370_MOESM3_ESM.pdf]

Description of Additional Supplementary Files

File Name: Supplementary Data 1

Description:
Description of information in each sheet:
Column Definitions – Definitions of the columns in the different sheets.

Mass_spectrometry setup: Biological replicate, SILAC label, treatment condition, and experiment type for mass spectrometry-based quantification of lysine acetylated (AcK) peptides and proteins.

ActD_NVP-2: List of AcK sites quantified after inhibition of transcription by ActinomycinD or NVP-2. P-values and log2 fold changes are from two-sided t-test.

ActD_NVP-2_CoreHistones: List of acetylated peptides quantified in core histones (H2A, H2B, H3, H4) after inhibition of transcription by ActinomycinD or NVP-2.

TRP: List of AcK sites quantified after inhibition of transcription by Triptolide.

TRP_CoreHistones: List of acetylated peptides quantified in core histones (H2A, H2B, H3, H4) after inhibition of transcription by Triptolide.

IF_intensities_H3K27ac: Immunofluorescence intensities of cells stained with H3K27ac after treatment with DMSO, ActD, NVP-2, Trp, A485.

IF_intensities_H2BK120ub: Immunofluorescence intensities of cells stained with H2BK120ub after treatment with DMSO, ActD, NVP-2, Trp, A485.

H3K27ac ChIP-seq peaks: Genomic position of H3K27ac ChIP-seq peaks and their regulation by ActD or NVP-2 treatment.

EU-seq changes after Trp tx: Nascent transcription changes, quantified by EU-seq, after triptolide treatment.

IF_intensities_H3K27ac: Immunofluorescence intensities of cells stained with H3K27ac (antibody: CST#8173) after treatment with DMSO, ActD, NVP-2, A485.
